# Risk of myocardial infarction and stroke after breast cancer: an analysis of a population of 1.3 million women from North-West Italy

**DOI:** 10.1007/s10549-026-07900-0

**Published:** 2026-01-29

**Authors:** Fulvio Ricceri, Enrica Favaro, Alberto Catalano, Gregory Winston Gilcrease, Sara Claudia Calabrese, Elisa Ferracin, Daniela Di Cuonzo, Alessandra Macciotta, Lucia Dansero, Angelo d’Errico, Pierfrancesco Franco, Gianmauro Numico, Roberto Gnavi, Giuseppe Costa, Eva Pagano, Carlotta Sacerdote

**Affiliations:** 1 Centre for Biostatistics, Epidemiology, and Public Health (C-BEPH), Department of Clinical and Biological Sciences, University of Turin, Turin, Italy; 2 Department of Medical Sciences, University of Turin, Turin, Italy; 3 Department of Translational Medicine (DIMET), University of Eastern Piedmont, Novara, Italy; 4 UNESCO Chair in Sustainable Development and Territory Management, University of Turin, Turin, Italy; 5 School of Medicine and Surgery, University of Turin, Turin, Italy; 6 Unit of Epidemiology, Regional Health Service, ASL TO3, Grugliasco, Turin, Italy; 7 Unit of Cancer Epidemiology, “Città Della Salute E Della Scienza” Hospital and Centre for Cancer Prevention, Turin, Italy; 8 Unit of Oncology, Santa Croce E Carle” Hospital and Regional Oncology Network, Cuneo, Italy; 9 Department of Health Sciences (DISS), University of Eastern Piedmont, Novara, Italy; 10 Unit of Epidemiology, Local Health Unit of Novara, Novara, Italy

**Keywords:** Myocardial infarction, Stroke, Breast cancer, Chemotherapy, Competing risks

## Abstract

**Purpose:**

Breast cancer (BC) is a leading public-health issue affecting women on a global scale. Thanks to the widespread implementation of screening programs and the improvement in therapies, women with BC live longer but they also are more likely to experience an increased risk of other diseases. Reasons for this increased risk include genetics, shared risk factors, and adverse effects from BC treatment. Therefore, this research aimed to analyse the risk of myocardial infarction (MI) and stroke in women with BC, considering the potential side effects of treatments.

**Methods:**

For the analysis, we used data coming from the Piedmont Longitudinal Study (PLS), an administrative cohort based on the record-linkage among census data and several health-administrative databases involving more than 4 million inhabitants. The study population comprised women aged 30–75 years from the PLS study, excluding those with myocardial infarction or stroke at baseline. To analyse the investigated associations, competing risk analyses were performed, through the Cause-Specific Proportional Hazards model.

**Results:**

Among 1,342,333 women ranging from 30 to 75 years old, 19,203 had a BC diagnosis, of whom 206 (1.1%) experienced a subsequent MI and 203 (1.1%) a stroke. Women with BC showed an increased risk for MI (HR: 1.20; 95% CI 1.05–1.38) and for stroke (HR: 1.58; 95% CI 1.38–1.82). Chemotherapy was observed to be the major risk factor for MI in BC women, while no different risk by therapy was found for stroke.

**Conclusion:**

The results supported the hypothesis about the toxic effect of BC therapies, suggesting that clinicians should routinely and actively screen for treatment-related toxicities in women with BC and that researchers should prioritize personalized treatments to minimize potentially devastating side effects.

**Supplementary Information:**

The online version contains supplementary material available at 10.1007/s10549-026-07900-0.

## Introduction

Breast cancer (BC) remains a leading public-health issue affecting women on a global scale [1]. In Europe, BC is the most frequently diagnosed cancer in women and its incidence remains high, with recent projections suggesting a stable-to-slightly increasing trend in several countries, including Italy [1–3].

Most of the clinical research in the last 40 years has prioritized diagnosis and treatment, leading to a wider availability of improved methods for diagnosis (such as screening and early detection programs) and more effective therapeutic options [3]. This has led to tangible improvement in the clinical outcome and consequently prognosis [4, 5]. Moreover, improvement in BC management has led to a significant downtrend in mortality and an upward trend in survival [4, 5].

However, as BC survival rates improve, there is a growing concern regarding the long-term health consequences faced by survivors. Patients with BC live longer following diagnosis but they also are more likely to experience an increased risk of other diseases, including second primary cancers [6, 7], cardiovascular diseases and metabolic diseases [8, 9], at least partially attributable to treatment-related toxicity [4]. In particular, cardiovascular and cerebrovascular events have gained increasing attention as major determinants of long-term morbidity and mortality among BC survivors, potentially driven by both shared risk factors and cancer treatment–related toxicity [8–12]. Notably, cardiovascular disease represents one of the leading non-cancer causes of death in long-term BC survivors, making the prevention and monitoring of major vascular outcomes a key survivorship priority [10].

Therapeutic management of BC depends on the type and stage of breast cancer at the time of diagnosis. Depending on the severity of the disease, on the tumour subtype and on the clinical characteristic and preferences of the patients, the guidelines for the treatment of BC include different types of treatments ranging from conservative surgery or radical mastectomy to radiotherapy, systemic chemotherapy or hormonal and biological therapy [1–3].

Breast cancer treatments can induce both early and late adverse effects, with late complications sometimes requiring months or years to develop and potentially affecting long-term morbidity and health-related quality of life [4]. Among these, cardiovascular and vascular toxicities are particularly relevant because they may contribute to atherosclerotic and thrombotic pathways beyond the acute treatment phase [11–13]. For instance, radiotherapy may increase long-term coronary risk in a dose-dependent manner, with effects that can begin within a few years and persist for decades, supporting a plausible mechanistic link with ischemic endpoints such as MI [8].

Among the most significant long-term complications, cardiotoxicity has emerged as a major concern due to its impact on survivors’ morbidity, quality of life, and overall mortality [11]. Indeed, cardiovascular toxicity is increasingly hypothesized to result not only from specific breast cancer treatments but from cancer therapies in general [11–13]. Thus, the field of cardio-oncology was established in response to this growing complexity of oncologic management [14]. In particular, the role of chemotherapy and radiotherapy on cardiac toxicity has been extensively studied [15]; however, most of these studies have been conducted in clinical settings with medium-short follow-up periods [16, 17] while only a few have focused on real-world population studies [18, 19].

Beyond myocardial dysfunction and heart failure, increasing attention has also been directed toward treatment-related vascular injury and thrombo-inflammatory mechanisms that may translate into major cerebrovascular events, including stroke, thereby expanding the cardio-oncology perspective to encompass the broader spectrum of cardiovascular and cerebrovascular outcomes [11–13].

A recent systematic review and meta-analysis summarizing the available observational evidence reported an overall increased risk of major cardiovascular outcomes among BC survivors, including both MI and stroke, compared with women without BC; however, estimates were heterogeneous across studies, reflecting differences in populations, follow-up duration and treatment exposures [20].

MI and stroke are clinically meaningful “hard endpoints” with major implications for survivorship and prevention, and they are generally well captured in administrative healthcare data, making them suitable outcomes for population-based investigations.

The aim of this study was to analyse the risk of cardiovascular and cerebrovascular diseases, particularly myocardial infarction (MI) and stroke, in women diagnosed with BC, with a particular focus on potential treatment-related effects in a large population study. To achieve this goal, we utilized administrative health data from the piedmont longitudinal study (PLS). Given the clinical relevance of MI and stroke as severe, well-defined outcomes with important implications for long-term survivorship, we specifically investigated these endpoints using a competing-risks framework to provide unbiased estimates in the presence of competing mortality.

## Methods

### Study design and participants

Women included in the study (are range 30 and 75 years old) are part of the Piedmont Longitudinal Study (PLS), an administrative longitudinal cohort that includes all the residents in the Piedmont region, Italy (approximately 4 million subjects), linked with the 2011 census data and followed-up through administrative record-linkage for mortality, hospital admissions, drug prescriptions, and specialist visits.

### Exposure and outcome definition

Using a validated algorithm [21], we identified all patients diagnosed with incident BC in Piedmont between January 1, 2011, and December 31, 2017 (exposure). Moreover, in the same period, we identified incident cases of MI and stroke, using the algorithms proposed in the National Outcome Plan (https://pne.agenas.it/) (outcomes).

Women with prevalent BC, MI, or stroke (identified in the period 2006–2010) have been excluded from the analyses.

Furthermore, from the combination of the aforementioned data, we identified the type of treatment (chemotherapy, radiotherapy, hormones) offered to our cohort of BC cancer patients.

### Other variables definition

The administrative nature of the study does not allow to identify other individual risk factors, such as hypertension, diabetes, body mass index (BMI), smoking, etc. However, some proxy variables have been computed using information available in health-administrative databases. Hypertension, hypercholesterolemia, and diabetes have been estimated considering the respective drug consumption. Socioeconomic position (SEP) was well assessed thanks to the linkage with census data and it was measured using education, occupation, and material living conditions [22]. Smoking status was impossible to retrieve, however we identified subjects with chronic obstructive pulmonary disease (COPD, using drug prescriptions and hospital discharges), considering this variable as a proxy for heavy smoking. No information for BMI and alcohol consumption was available.

### The EPIC-Turin study

To overcome the aforementioned problem of no information about confounding variables, a sensitivity analysis was conducted in the EPIC-Turin study, whose 10,604 participants (4,558 women) were part of the PLS. The EPIC-Turin study was described elsewhere [23]. Briefly, healthy volunteers aged 30–74 have been recruited between 1993 and 1998 in the Turin municipality (Piedmont, Italy). All subjects filled a detailed lifestyle and dietary questionnaire and provided a blood sample that was stored in liquid nitrogen. Subjects have been followed up for mortality, cancer incidence, cardiovascular and cerebrovascular disease incidence, and diabetes until the 31st December 2012. In this cohort, information at baseline about hypercholesterolemia, hypertension, diabetes, smoking status, BMI, alcohol consumption, and SEP are available.

### Statistical analysis

The study population was described using frequency and percentage for qualitative variables and mean and standard deviation (SD) and/or median and interquartile range (IQR), for quantitative variables. The normality of quantitative variables was tested through the Kolmogorov–Smirnov test. Participants with missing data for key variables were excluded from the analyses.

In this study, subjects were followed from 1st January 2011 (starting point for the whole population) to 31st December 2017. Women who developed BC were considered as exposed starting from the date of BC diagnosis. The follow-up period ended at the earlier of the following dates: incidence of the first MI or Stroke, death, lost to follow-up, or end of follow-up period. Crude rates were calculated using the following formula: $$\frac{\# new cases from 01.01. 2011 to 31.12. 2017}{person-years whole population \mathrm{01.01.2011}- \mathrm{31.12.2017}}$$. Regarding the effect of BC diagnosis on the risk of developing MI and stroke, it was evaluated by conducting a competing risks analysis, a particular statistical method that is part of the Survival analysis. Specifically, we fitted different Cause-Specific Proportional Hazards models, considering the death from causes other than MI or stroke as competing event and estimating the Cause-Specific Hazard Ratio (HR) and related 95% confidence interval (CI). All models were conducted using two different adjustments: one by age at baseline and the other by the described confounding variables. Firstly, BC women were compared with healthy women, then women who undertook a specific therapy were compared both with healthy women and with BC patients who have had neither chemotherapy nor radiotherapy.

In addition, we considered women that were at least 55 years old at baseline (as a proxy of a subset of postmenopausal women). Moreover, we considered as outcomes both haemorrhagic and ischemic stroke. Finally, we compare women with treated with aromatase inhibitors or tamoxifen with women with BC not treated with those therapies.

Finally, we performed a sensitivity analysis, comparing the differences in the Cause-Specific Hazard Ratio estimates between models with minimal and fully adjustment in PLS and in EPIC-Turin to infer a possible overestimation due to incomplete adjustment in PLS.

STROBE checklist is presented in Supplementary Table 1.

## Results

The population observed in this study is composed of 1,342,333 women aged 30 to 75 years, and residents in the Piedmont region on 1 January 2011, with 19,203 having a diagnosis of BC in a median follow-up time of 6.9 (IQR: 6.9–6.9) years. Description of the population is presented in Table 1. Women with BC are older, with more prevalent hypercholesterolemia, hypertension, diabetes, and COPD then their counterpart, possibly due to their older age.

**Table 1:** Baseline characteristics of the women (age 30–75) included in the study and of those women with breast cancer (BC)

	General population (women) *N* = 1,342,333	Women with BC^a^ N = 19,203
*Age*		
Mean (St Dev)	51.81 (12.78)	60.04 (11.35)
*COPD*^b^		
N (%)	70,061 (5.2%)	1,159 (6.0%)
*Hypercholesterolemia*		
N (%)	106,456 (7.9%)	2,035 (10.6%)
*Diabetes*		
N (%)	53,775 (4.0%)	902 (4.7%)
*Hypertension*		
N (%)	338,941 (25.2%)	6,272 (32.7%)
*Marital status*		
Married N (%)	843,881 (62.9%)	12,571 (65.5%)
Single N (%)	229,532 (17.1%)	2,399 (12.5%)
Separated, divorced, widowed N (%)	267,854 (20.0%)	4,229 (22.0%)
*Education*		
*Degree N (%)*	168,493 (12.6%)	2,077 (10.8%)
Secondary school N (%)	336,752 (25.1%)	4,352 (22.7%)
Middle school N (%)	552,663 (41.2%)	8,020 (41.7%)
*Primary*		
School or none N (%)	283,358 (21.1%)	4,750 (24.8%)
*Employment status*		
Employed N (%)	674,549 (50.3%)	8,130 (42.3%)
Unemployed N (%)	53,769 (4.0%)	517 (2.7%)
Housewife N (%)	240,400 (17.9%)	3,433 (17.9%)
Retired or other N (%)	372,544 (27.8%)	7,119 (37.1%)
*Material living condition*		
Good N (%)	1,151,666 (85.8%)	17,122 (89.2%)

^a^*BC* Breast cancer; ^b^*COPD* Chronic obstructive pulmonary disease

Among women without a breast cancer diagnosis during the follow-up period, 32,273 (2.4%) developed a competing event (death) by the end of the observation period, whereas 944 (4.9%) died among women who developed BC.

Focusing on BC patients, 7,660 patients were treated with radiotherapy only, 1,992 patients were treated with chemotherapy only, 5,832 patients were treated with both radiation therapy and chemotherapy, and 3,719 patients were not treated with radiotherapy nor with chemotherapy. Moreover, 14,767 women were treated using at least one aromatase inhibitor (letrozole, anastrozole or exemestane) and 10,752 were treated with tamoxifen (they may also receive other treatments besides tamoxifen).

Description of baseline characteristics of the women with BC who underwent radiotherapy or chemotherapy are presented in Supplementary Table 2, while the numbers of women with breast cancer who received different treatment combinations and experienced (or not experienced) an event are shown in Table 2.

**Table 2:** Numbers of women with breast cancer who received different treatment combinations and experienced an event

	Women with BC (*N* = 19,203) N (%)	Myocardial Infarction (*N* = 206) N (%)	Stroke (*N* = *203*) N (%)
Women with BC^a^ who underwent radiotherapy only	7,481 (39.0%)	88 (42.7%)	91 (44.8%)
Women with BC^a^ who underwent chemotherapy only	1,938 (10.1%)	37 (18.0%)	17 (8.4%)
Women with BC^a^ who underwent both radiotherapy and chemotherapy	5,739 (29.9%)	49 (23.8%)	44 (21.7%)
Women with BC^a^ who have had neither chemotherapy nor radiotherapy	4,045 (21.1%)	32 (15.5%)	51 (25.1%)
Women with BC^a^ treated with at least one aromatase inhibitor	14,465 (75.3%)	147 (71.4%)	155 (76.4%)
Women with BC^a^ treated with tamoxifen	10,480 (54.6%)	130 (63.1%)	142 (70.0%)

^a^*BC* Breast cancer

### Risk of MI and stroke in women with BC

Of the women with BC, 206 had MI (crude rate: 2.92 per 1000 person-years) and 203 experienced stroke (crude rate: 2.85 per 1000 person-years), whereas among the women without BC, 19,921 had MI (crude rate: 2.20 per 1000 person-years) and 16,378 experienced stroke (crude rate: 1.81 per 1000 person-years).

Women with BC showed an increased risk for both MI and stroke compared to women without BC. The difference was statistically significant for both models with minimal and fully adjustment (Supplementary Table 3). In the model with fully adjustments, women with BC showed an increased risk for MI (HR: 1.20; 95% CI 1.05–1.38) and for stroke (HR: 1.58; 95% CI 1.38–1.82) with respect to women without BC.

### Risk of MI and stroke in women with BC, according to different therapies

In our analysis, we observed an increased risk of stroke of approximately 50% in women with breast cancer compared to women without breast cancer, independently of the treatment received. Specifically, women who underwent radiotherapy only had a not-significant increased risk of MI (HR = 1.12; 95% CI 0.91–1.38) while those who received chemotherapy only had significant increased risk of MI (HR = 2.60, 95% CI 1.89–3.60). Moreover, considering the risk of stroke, women who underwent radiotherapy only had a HR of 1.57 (95% CI 1.28–1.93) and those who received chemotherapy only had a HR of 1.66, (95% CI 1.03–2.67). Results could be found in Table 3.

**Table 3:** Results of the Cox model analysing the association between breast cancer development and the risk of myocardial infarction and stroke, accounting for therapies

	Myocardial Infarction HR^a^ (95% CI^b^)^c^	Stroke HR^a^ (95% CI^b^)^c^
*Comparison: women without breast cancer*		
Women with BC^d^ who underwent radiotherapy only	1.12 (0.91 – 1.38)	1.57 (1.28 – 1.93)
Women with BC^d^ who underwent chemotherapy only	2.60 (1.89 – 3.60)	1.66 (1.03 – 2.67)
Women with BC^d^ who underwent both radiotherapy and chemotherapy	1.09 (0.82 – 1.44)	1.34 (0.99 – 1.80)
Women with BC^d^ who have had neither chemotherapy nor radiotherapy	0.95 (0.67–1.35)	1.88 (1.42–2.47)
*Comparison: women with breast cancer who have had neither chemotherapy nor radiotherapy*		
Women with BC^d^ who underwent radiotherapy only	1.18 (0.79 – 1.77)	0.80 (0.56 – 1.14)
Women with BC^d^ who underwent chemotherapy only	2.65 (1.64 – 4.28)	0.85 (0.49 – 1.49)
Women with BC^d^ who underwent both radiotherapy and chemotherapy	1.14 (0.73 – 1.80)	0.67 (0.44 – 1.02)

^**a**^*HR* hazard ratio; ^b^*CI* confidence interval; ^c^Models are adjusted by age, chronic obstructive pulmonary disease (COPD), hypercholesterolemia, diabetes, hypertension, marital status, education, employment status, and material living condition; ^d^*BC* Breast cancer

However, when comparing women with breast cancer who underwent specific therapies to those with breast cancer who did not receive radiotherapy or chemotherapy, no increased risk of stroke was observed.

### Subgroup analyses

Women with BC treated with aromatase inhibitors or tamoxifen did not show an increased risk of MI (HR: 0.78; 95% CI 0.58–1.04 and HR: 0.82; 95% CI 0.56–1.20, respectively) compared with women with BC not treated with these drugs, while a significant increase of stroke was observed for those BC women treated with aromatase inhibitors (HR: 0.97; 95% CI 0.71–1.32 and HR:0.76; 95% CI 0.51–1.14) compared with women with BC not treated with these drugs.

When analysing the subset including postmenopausal women only, the association between BC vs non-BC and MI (HR: 1.19; 95% CI 1.03–1.38) and stroke (HR: 1.54; 95% CI 1.33–1.78) remained significant and results did not differ substantially from those obtained in the main database for all the investigated BC therapies (Supplementary Table 4).

Women with BC had a similar higher risk of haemorrhagic and ischemic stroke compared to healthy women (HR: 1.83; 95% CI 1.40–2.38 and HR: 1.50; 95% CI 1.26–1.77, respectively) and the same similarity occurred for all the type of therapies investigated (Supplementary Table 5).

### Sensitivity analyses

To estimate if a full adjustment could have changed the results, we measured the risk of MI and stroke in the EPIC-Turin cohort (descriptive statistics about the characteristics of the cohort were reported in Supplementary Table 6), where the number of women with BC and a subsequent MI or stroke was very low (n = 4) due to the initial size of the cohort. However, even without statistical significance, results are very close for the minimal (HR 2.52; 95% CI 0.90–7.05) and the fully adjustment (HR 2.54; 95% CI 0.90–7.15), supporting the hypothesis that the residual confounding in the main analyses, if present, is low.

## Discussion

In this large population-based study, we observed that women diagnosed with BC had an increased risk of major vascular outcomes, with distinct patterns for myocardial infarction and stroke. Specifically, MI risk was higher among women receiving chemotherapy and, to a lesser extent, radiotherapy, with the strongest association observed when chemotherapy was administered as a standalone treatment. In contrast, although stroke risk was increased in women with BC compared with women without BC, this excess risk did not appear to be explained by specific treatments, as differences across therapy categories were not evident. Overall, these findings provide real-world evidence on clinically meaningful cardiovascular and cerebrovascular endpoints in BC survivors using a competing-risks framework, which is essential in the presence of competing mortality.

Cardiovascular disease is one of the leading causes of morbidity and mortality among BC survivors [24], and treatment-related toxicity is considered a major contributor to this long-term burden. In different clinical settings, many forms of chemotherapy and radiation have been associated with increased risks of cardiovascular complications, including coronary artery disease and thromboembolic and cerebrovascular events [9, 11–13, 25, 26]. However, the magnitude and specificity of these associations, particularly for “hard endpoints” such as MI and stroke, remain variable across studies, highlighting the value of population-based evidence.

Our study, which included a large number of participants, confirmed previous findings in the literature by showing that chemotherapy is a strong risk factor for MI. These findings are consistent with the broader literature indicating that systemic anti-cancer therapies may adversely affect cardiovascular health through multiple pathways, including direct myocardial injury, endothelial dysfunction, oxidative stress, and pro-thrombotic mechanisms [27, 28]. Studies on BC confirm the higher risk of cardiovascular disease among chemotherapy treated patients [14, 18, 29–31]. In BC treatment, the traditional third generation polychemotherapy regimens are the most widely used treatment and consist of anthracycline and taxanes administered sequentially [2]. Anthracyclines such as daunorubicin and mitoxantone are the most well-studied and generate dose dependent cardiomyopathy and congestive heart failure with a possible mechanism involving reactive oxygen species (ROS) generation and topoisomerase 2 [27, 28, 32–35]. Additionally, taxanes such as paclitaxel and docetaxel can primarily cause arrhythmias, bradycardia and myocardial ischemia [36, 37]. Overall, these mechanisms provide biological plausibility for an increased risk of ischemic outcomes following chemotherapy exposure, although the magnitude and timing of events may vary across agents and patient profiles [27, 28].

Similar to chemotherapy, radiotherapy is also associated with a variety of cardiovascular complications involving the pericardium, myocardium, valves, coronary arteries and conduction systems [11]. Radiotherapy irradiation of the heart has been shown to be associated with long-term cardiac toxicity such as heart failure, coronary artery disease, myocardial infarction, and cardiovascular death [33, 38]. At a cellular level radiotherapy effects seem to induce cardiac injuries mediated by reactive oxygen species and myocardial fibrosis [39]. The risk of cardiovascular damage due to radiotherapy seems to be higher especially in left-sided cancers that generally receive a higher dose of radiation to the heart than those with right-side irradiation, as well as in women with pre-existing cardiac risk factors [8, 33, 38]. Among the past epidemiological studies there are a number of different results on the contribution of radiotherapy to cardiovascular events [38]. The different results between studies could be probably due to the variety of radiation therapy regiments in the history of BC: indeed, the range of doses to the heart has changed over the past few decades [40].

In addition to cardiac effects, radiotherapy may also contribute to cerebrovascular risk in specific settings, for example when nodal irradiation results in relevant exposure of large cervical vessels [41]

In our study, radiotherapy showed a smaller association with MI risk than chemotherapy, which may reflect both differences in underlying mechanisms and improvements in contemporary radiation delivery techniques.

A recent analysis by the Danish Breast Cancer Group reported on a 10-year cumulative risk of cardiac event of 1–2% (1.8–2.4) for left-sided breast cancer patients, irradiated with computed- tomography based radiotherapy with an incidence ratio of 0.9 (0.69–1.16) [42]. This evidence is aligned with the findings that modern techniques of radiotherapy are designed to minimize exposure of the heart and vasculature [43, 44]. In the last 20 years, new possibilities emerged in breast cancer RT allowing for targeted solutions and personalized approaches, such as increased adoption of hypofractionation, selective use of the boost to the lumpectomy cavity, reduction in treatment volume with partial breast irradiation, introduction of volume-based target volume definition and selection, integration with primary systemic therapy strategies, decreased the treatment-related toxicity profile in order to reduce the harm to the cardiovascular system [45].

Our research showed that both chemotherapy and radiotherapy for BC are associated with an increased risk of MI, even if it is significant for chemotherapy only and this effect becomes even more pronounced when chemotherapy is administered as a standalone treatment. The lack of a clear synergistic effect between chemotherapy and radiotherapy on MI risk may have several explanations.

One possible explanation is patient selection bias: patients receiving chemotherapy alone may have different baseline characteristics compared to those undergoing combined treatment. For instance, they may have more comorbidities or preexisting cardiovascular risk factors, making them inherently more susceptible to MI. Additionally, oncologists might avoid radiotherapy in patients with a higher cardiovascular risk profile, leading to a concentration of these high-risk individuals in the chemotherapy-only group [46]. Another possible explanation is the difference in the timeline of cardiovascular toxicity between the two treatments. Radiation-induced cardiovascular damage typically develops over a longer period, whereas chemotherapy-related effects on the cardiovascular system may be more acute. As a result, in studies with shorter follow-up periods, the impact of chemotherapy on MI risk may appear more pronounced, while the long-term cardiovascular effects of radiotherapy might not yet be fully evident [47]. Finally, heterogeneity in treatment indications and sequencing, as well as unmeasured clinical factors related to tumour severity and patient fitness, may further contribute to differences across therapy categories[46, 47]

Furthermore, the results of our research revealed that the cardiotoxic effects of chemotherapy and radiation are distinct from any potential cardiotoxic effects of adjuvant treatments. Indeed, our results showed that different hormonal therapy (both tamoxifen and aromatase inhibitors) were not clearly associated with on the risk of MI. Recently, a number of studies considered the effects of adjuvant therapies, in particular of tamoxifen and aromatase inhibitors on cardiovascular risk, but the existing studies are still conflicting, hence supplementary research is needed [48, 49]. In our setting, these findings should be interpreted cautiously and primarily as descriptive, because administrative data do not allow a fully detailed characterization of endocrine therapy exposure and because an internal comparison within the BC cohort would be required to isolate therapy-specific effects.

Finally, our study wanted to investigate the risk of stroke after treatment for BC. While there are a number of studies evaluating the risk of MI, evidence of an association between treatment for BC and stroke is limited [29, 41]. Prior studies have found increased risk of stroke in patients with BC who were given radiotherapy [41]. More specifically it has been found that radiation to the supraclavicular lymphnodes gives a significant dose of radiation to the proximal carotid artery, which increases the risk of carotid stenosis and ischaemic stroke [41].

Our data indicated that women with BC showed an increased risk for stroke compared to women without BC, however the risk for stroke seems to be independent from specific treatments, including both chemotherapy and radiotherapy. Indeed, the higher risk for stroke observed comparing BC (any therapy) with women without BC disappeared when comparing therapies among BC patients.

The results are the same considering both haemorrhagic and ischemic stroke. This data validates other studies that do not detect any association between stroke risk and specific chemotherapy regimens [29]. It was known that MI and stroke share a lot of common risk factors but our intriguing results suggest that in the pathogenesis of stroke other different factors could be involved indirectly as consequences of tumour effects [50]. Further studies could clarify if genetic susceptibility, as well as other factors such as stress, smoking, and hypertension, could determine a higher risk of stroke independent from specific therapies [29]. In summary, the elevated stroke risk in women with breast cancer is likely due to factors inherent to the disease itself, such as shared risk factors, cancer-induced hypercoagulability and systemic inflammation. These factors contribute to the increased stroke risk independently of specific cancer treatments, explaining why the higher risk observed when comparing BC patients to women without BC disappears when comparing different therapies among BC patients.

Although this study was conducted in a single Italian region, the Piedmont cohort is large and population-based, and breast cancer management broadly follows national and international treatment guidelines. Therefore, we expect the overall direction of the associations—particularly the increased MI risk observed among women treated with chemotherapy—to be broadly generalizable to similar healthcare settings. However, absolute risks and the magnitude of associations may differ across populations due to variation in baseline cardiovascular risk profiles, comorbidity burden, screening uptake, and treatment protocols, as well as follow-up duration.

Even with a number of intriguing findings, the present study has several potential limitations.

The main limitation of the study is that we cannot identify patients with tumours on the left side and on the right side, as cardiac radiation doses are higher on the left side in BC patients with left-sided tumours. Moreover, it was not possible to obtain information on the type, cumulative dose, or intensity of chemotherapy used. This limitation is relevant because cardiovascular and vascular toxicity profiles can differ substantially across chemotherapy classes and may be dose-dependent, and the lack of this information may have introduced exposure heterogeneity within the broad “chemotherapy” category, limiting treatment-specific interpretation and preventing dose–response evaluations. In addition, information on breast cancer severity (e.g., stage and grade) was not available in the administrative data, and therefore we could not adjust for disease aggressiveness. This may be important because more severe disease is more likely to be treated with systemic therapies and may also be associated with an increased risk of vascular outcomes through disease-related mechanisms, potentially contributing to differences observed across therapy categories. Another important limitation is that there isn’t adjustment for some important factors such as BMI and alcohol consumption, both associated with BC and MI and therefore possible confounders. However, the adjustment for socioeconomic factors mitigates the risk of bias due to the missing information for those variables. Moreover, we used the EPIC-Turin study to evaluate the amount of residual confounding and, even if the sample size in this cohort is small, confounding seemed to be small, if present. Additionally, although this is one of the largest population studies conducted, the simultaneous occurrence of BC and CVD in the same woman remains a rare event, resulting in a limited number of cases for subgroup analyses. Finally, the short follow-up period of this study, from 2011 to 2017, is a significant limitation because the late effect of therapies could need more time to show its damage. In addition, given the observational nature of the study, our findings reflect associations and should not be interpreted as evidence of causation. Nevertheless, this study is one of the largest studies on this topic, based on the entire population of Piedmont region (more than 4 million inhabitants), where nearly 20,000 women with BC have been found in the follow-up period.

## Conclusion

In conclusion, in this large population-based cohort, breast cancer survivors, particularly those receiving chemotherapy, showed an increased risk of myocardial infarction, whereas the excess risk of stroke appeared less clearly attributable to specific treatment categories. These results support the importance of integrating cardiovascular risk assessment and monitoring into breast cancer survivorship care and of considering vascular outcomes when planning follow-up strategies. However, given the relatively short follow-up available in our study, longer longitudinal investigations with more detailed treatment information are needed to better characterize late-onset effects, especially those potentially related to radiotherapy.

## Supplementary Information

Below is the link to the electronic supplementary material.

Supplementary file1 (DOCX 38 KB)

## Data Availability

Raw data cannot be made freely available due to restriction imposed by the National Statistics Plan which do not allow open/public sharing of data on individuals. However, aggregated data are available upon request. Requests should be sent to the corresponding author.
